# Emergency Department-initiated Naltrexone for Alcohol Use Disorder: Association with Recidivism

**DOI:** 10.5811/westjem.52971

**Published:** 2026-07-16

**Authors:** Otis Warren, Jeremiah Gress, Timmy Lin, Melanie Lippmann, Gary Bubly

**Affiliations:** Brown University Health, Department of Emergency Medicine, Providence, Rhode Island

## Abstract

**Introduction:**

Alcohol use disorder (AUD) contributes to significant healthcare costs, disease, emergency department (ED) crowding, and recidivism. Naltrexone is a treatment for AUD that has been approved by the U.S. Food and Drug Administration and holds promise as a pharmacologic intervention for ED patients with AUD. We hypothesized that subjects who received naltrexone in the ED would have associated reduced recidivism in the 30 days after treatment.

**Methods:**

This was a retrospective chart review of an AUD quality program comparing ED visits in the 30 days before with 30 days after initiation of naltrexone in the ED. We performed this study at two academic hospitals (one a Level I trauma center) and one community hospital.

**Results:**

A total of 297 subjects (median age 43 years, 69.5% male) made 331 naltrexone index visits during the study period. There was no difference in the number of ED visits before versus after the index visit among all subjects (0 median visits before [interquartile range 0–1] versus 0 median after [0–1]). Subjects who were frequent ED users (defined as ≥ 4 ED visits in the 90 days preceding (N=56 [16.9%]) had a significant reduction in ED visits after administration of naltrexone (median of 5 visits [3–8] before versus 3 [2,6] after), with 36 of these subjects (64.3%) having reduced ED visits in the 30 days after naltrexone administration.

**Conclusion:**

Naltrexone administered in the emergency department had no associated change on the study population’s rates of recidivism for alcohol use disorder, but it was associated with reduced ED recidivism in subjects with high ED use.

## INTRODUCTION

Alcohol use disorder (AUD) contributes significantly to disease burden, healthcare costs, hospitalization, and resource use for emergency departments (ED) throughout the United States. It is estimated that alcohol accounts for $249 billion in healthcare costs annually with heavy episodic drinking accounting for most of that cost at $191.1 billion. 1–3 Additionally, patients with AUD are more likely to be frequent users of the ED and emergency medical services. 1,4 Providing effective treatment to patients with AUD in the ED is difficult due to limitations of clinicians’ understanding of pharmacotherapy options available, the lack of continuity of care, time constraints, and costs. 5–7

Naltrexone is approved by the U.S. Food and Drug Administration (FDA) for treating AUD. Naltrexone is an opioid receptor antagonist, thought to reduce the opioid-mediated pleasure response from ethanol use. 8,9 Randomized studies in outpatient settings of naltrexone for AUD have shown mixed results with trends toward improvement in reduction in heavy drinking days, particularly when combined with behavioral therapy. 10–13 This poses great potential for benefit in patients presenting to the ED with AUD and, more specifically, in those who report “binge-drinking.” A limited number of feasibility studies have demonstrated increases in quality of life and reduction in alcohol cravings for patients starting naltrexone in the ED, suggesting the need for further study. 14–16 There have been no studies evaluating naltrexone’s effect on ED visits and recidivism. In this study, we evaluated a quality program with insurers to provide naltrexone to patients with suspected AUD in the ED. Our hypothesis was that subjects who received ED-initiated naltrexone as part of the quality program would have associated reduced recidivism.

## METHODS

We performed a retrospective chart review of naltrexone in an AUD quality program. Our primary outcome was ED visits by subjects with suspected AUD in the 30 days prior to ED-initiated naltrexone compared to ED visits in the 30 days following. Secondary outcome measures were a subject’s visits with non-high ED use (defined as fewer than four ED visits in the preceding 90 days) and high ED use (more than four ED visits in the preceding 90 days).

### Naltrexone Quality Program

In July 2022, a quality program was developed between the hospital system and multiple insurers. After outreach, four of the major commercial and managed Medicaid insurers in our catchment area agreed to contract with the healthcare system to create a separate contractual carveout in addition to previously negotiated facility fees to cover the ED cost of extended-release (intramuscular) naltrexone (XR-NTX) to ED patients with suspected AUD. These insurers agreed to continue the program indefinitely with annual review. To date, none of the insurers have discontinued the contractual carveout. The clinical program was offered to patients with suspected AUD regardless of insurance status. The quality program was carried out at three hospitals within the same hospital system: 1) an urban, academic teaching hospital and Level I trauma center with an approximate annual ED volume of 138,000; 2) a community teaching hospital in the same city with an approximate annual ED volume of 70,000; and 3) a community hospital in a neighboring city with an approximate annual ED volume of 38,000.

Multiple educational lectures and resources were provided to attendings, residents, nurse practitioners, physician assistants, nurses, and pharmacists. This included lectures at residency conferences, faculty meetings and nursing huddles. Information was provided via slides with a summary of the evidence for naltrexone for AUD with attention paid to contraindications, particularly the risk of precipitated opiate withdrawal (see below). Follow-up emails to the above groups reiterated this information throughout the study period. Treatment with naltrexone was offered at the discretion of the clinician and the patient.

A chart flag was created alerting clinicians to consider offering naltrexone to patients with AUD. This alert flagged charts of patients who had at least one diagnosis within the health system of an alcohol-related illness within the prior five years. This flagged any patient who had an *International Classification of Diseases, 10^th^ Rev*, (*ICD-10*) code category F-10, which is a broad category defined as mental and behavior disorders due to use of alcohol.^17^ The alert—a banner that appeared on the side of the patient’s char—was visible upon first opening the chart and again in the disposition screen. It was not a “hard stop” flag and did not require physicians and advanced practice clinicians to interact with it. Clicking on the banner led to links to a standardized order set with safety measures and contraindications to naltrexone, as well as instructions for patient follow-up.

**Population Health Research Capsule**

What do we already know about this issue?

*Alcohol-related complaints represent a large proportion of emergency department (ED) and healthcare costs. Naltrexone shows promise in reducing heavy drinking*.

What was the research question?

*Is initiating naltrexone in the ED associated with reduced ED use?*

What was the major finding of the study?

*64.3% (n = 36/56) of subjects with high ED use made fewer visits after naltrexone initiation (P < .001)*.

How does this improve population health?

*Initiating naltrexone in the ED is associated with reduced ED use by patients with alcohol use disorder*.

An order set standardized the dosing and safety components. All subjects were given outpatient follow-up in an addiction medicine clinic located at one of the study site hospitals. Referrals were made electronically, and appointments were available to subjects within 1–3 days. The clinic also allowed for “drop-in” visits without appointments during business hours. Community health workers facilitated follow-up and patient outreach.

The main contraindication to receiving naltrexone was current opiate use. Given the potential for precipitated opiate withdrawal, patients were screened with a comprehensive history, medication dispense reports, and urine toxicology screens. For the patients receiving XR-NTX, a test dose of either 0.4 mg intravenous naloxone or 25 mg oral naltrexone was given prior to a full dose, and they were monitored for signs of opiate withdrawal. This was included in the order set.

Additional contraindications were allergy to naltrexone, pregnancy, age < 18 years, upcoming surgery within 30 days, and admission to inpatient on the index visit. Patients were excluded from XR-NTX administration if they had a history of Child-Pugh class III liver disease, or serum aspartate aminotransferase or alanine aminotransferase levels > 5 times above normal.

The decision to offer naltrexone to patients was at the discretion of the treating physician or advanced practice clinician. Patients who were receptive to starting naltrexone in the ED were offered either oral naltrexone or XR-NTX. The patient chose the specific formulation of naltrexone with counseling from the physician or advanced practice clinician. Patients were informed that XR-NTX would have a duration of action of 30 days and had the benefit of neither having to fill a prescription nor take a daily medication but was associated with pain at the injection site. Subjects received either 380 mg of intramuscular microsphere naltrexone (30-day duration of action) or 50 mg oral naltrexone in the ED with a 30-day prescription for 50 mg of once-daily oral naltrexone. Subjects were counseled to take this once daily either in the morning or before they would typically start consuming alcohol.

Upon discharge, patients were given standardized instructions on how to take the medication, safety precautions, and were advised that opiates would be less effective during naltrexone treatment. All patients were referred to the comprehensive addiction medicine clinic. Concomitant treatment for potential alcohol withdrawal was at the discretion of the treating physician.

### Data Collection and Analysis

This retrospective chart review of the quality metric was approved by the hospital’s institutional review board with a waiver of consent (IRB protocol number 2152433–6). We abstracted data from all subjects who received naltrexone in the ED between July 1, 2022–November 1, 2024. The single abstractor was blinded to the hypothesis. A standard data abstraction form was used. Subject data was deidentified after abstraction.^17^

We excluded subjects’ index visits from analysis if the subject was admitted to the hospital on the index visit or within the following 30 days, or if they received naltrexone in the 30 days prior to the index visit. Demographic data, modality of naltrexone used (XR-NTX versus oral), and 30-day rate of ED visit prior and following the index naltrexone visit were recorded (Table 1). All ED visits were considered, not just those related to alcohol use. Frequent ED users were defined as having four or more ED visits in the 90 days preceding the index naltrexone visit.

We used *t*-tests for analysis of age data (Tables 1 and 3). Chi-squared tests were used for analysis of demographic data and impact of naltrexone index visit (Tables 1–3). We used the Wilcoxon signed-rank test for statistical comparisons of 30-day prior and 30-day following data (Table 2).

## RESULTS

During the study period, the chart flag banner “fired” for 37,320 patient encounters across the study sites. The number of patients who were approached about initiating naltrexone was not recorded. A total of 297 subjects made 331 index visits (median age 43 years, 69.5% male) during which naltrexone was given (26 subjects had more than one index visit during the study period). During 144 index visits, patients received XR-NTX, and during 187 visits patients received the oral formulation. Those with frequent ED use were significantly older than those who presented to the ED less frequently; otherwise, there were no significant demographic differences between the groups (Table 1).

Table 2 shows the number of visits 30 day prior to the naltrexone index visit and the number of visits in the 30 days following the naltrexone index visit in all index visits, those with non-high use and those with high use. There was no significant difference between visits prior to and following the naltrexone index visit among all visits. Visits by subjects who did not have frequent ED use increased significantly after the naltrexone index visit, while visits by subjects with frequent use decreased significantly after the naltrexone index visit.

Table 3 shows the association of the index naltrexone visit on subsequent ED visits. Most (64.3%) subjects who had high ED use decreased their ED visits in the following 30 days. Most subjects who did not have high ED use had no change in their ED visits (69.5%)

## DISCUSSION

This is the first study to evaluate the association of naltrexone administration with rates of ED recidivism in patients with AUD. Although we found no difference in ED recidivism among all subjects, the majority of subjects with high ED use (64.3%) had reduced visits in the following 30 days. We also found a smaller but still significant increase in recidivism in subject visits with less frequent ED use, although most of these subjects had no change in the number of visits (69.5%).

Alcohol use disorder impacts patients’ health as it burdens the healthcare system more than any other substance use disorder. 1,3,4 In the ED, patients intoxicated with alcohol are frequently screened for concomitant illness and observed for safety monitoring without addressing their AUD, often to return again.^19^–^21^ Emergency departments are not the ideal place for comprehensive treatment of AUD, and an effective medication-based treatment is needed.

Administration of naltrexone in the ED holds promise for patients with AUD. Previous studies in ambulatory settings have shown reductions in alcohol consumption with naltrexone, particularly when combined with comprehensive support.^10^–^12^,^22^ Inpatient initiation of naltrexone has also shown similar reductions in alcohol consumption.^23^ In the past five years, naltrexone’s role in treating AUD in EDs has been explored. Several ED feasibility studies have shown moderate success in reduction of alcohol consumption and improvement in quality of life. 14–16 These studies did not evaluate the impact of naltrexone on healthcare use or ED visits. Our study adds to this growing body of literature and suggests that patients with frequent ED use and AUD may have associated reduced recidivism when treated with naltrexone.

The important unanswered question for future research is this: Does ED-initiated naltrexone reduce healthcare expenses incurred by alcohol use-related disease? Our study shows an association between naltrexone and reduced recidivism in patients with high ED use but ultimately does not answer this question. Several studies using economic modeling have suggested that naltrexone is a cost-effective intervention for AUD.^24^–^26^ One retrospective study demonstrated that outpatient medication use for AUD leads to lower healthcare costs for insurers even with more expensive formulations, such as XR-NTX.^27^

However, the practicalities in clinical practice are complicated; XR-NTX has a retail cost of approximately U.S. $1,500 per monthly dose and typically requires insurance pre-authorization, potentially limiting its practicality in EDs. Recent data does suggest an increasing number of states are passing laws that limit health insurer’s prior authorization for opiate use disorder and AUD treatment.^28^,^29^ Oral naltrexone also requires daily adherence. Both treatments require follow-up in addiction medicine clinics or primary care clinics that are comfortable continuing these therapies. In this ED-based study of naltrexone for AUD, a system-wide treatment plan and coordination with pharmacy, insurers, and addiction medicine was developed. Collaborative efforts such as this model may lead to increased naltrexone use for AUD and further reduction in costly ED use for AUD.

Future studies are also needed to determine who will benefit most from ED-initiated naltrexone. Our study showed an associated reduced recidivism only in those subjects with high ED use, and a trend toward increased recidivism among subjects with less frequent ED use. And even among our subjects with high ED use, 23.3% increased their ED use. We chose a 30-day follow-up window because this was the duration of the prescription for oral naltrexone given and the approximate duration of action of XR-NTX. A regression to the mean after the 30-day follow-up in this study was possible. Future studies should evaluate long-term outcomes, as all ED-based studies (including this one) have used short-term end points. Prospective cost effectiveness should also be studied in a clinical setting rather than via theoretical models.^24^,^26^ If naltrexone is deemed cost effective, the incentive for hospitals and insurers to develop robust naltrexone-based AUD treatment protocols could provide great benefit.

## LIMITATIONS

This study has many limitations. Firstly, because this was a retrospective chart review of a quality measure using naltrexone for AUD in the ED, any findings can only imply association and not causality. Many cofounders beyond the naltrexone itself could have contributed to the findings. Simply having the program and the departmental educational materials themselves could have led to better engagement with patients with AUD beyond naltrexone’s effect. Likewise, details about the subject’s alcohol use and quality of life were not collected. We also chose all ED visits, not just alcohol-related visits purposefully because of limitations in determining the reason for visit through a retrospective chart review. Patients were offered naltrexone at the discretion of the treating physician, and we do not know how many patients were offered naltrexone but declined. This factor also limits the standardization of the protocol and introduces biases with patient selection. Neither did we know the adherence rates in the subjects who were treated with the oral naltrexone.

This study was not designed to track adverse events and safety. While naltrexone is FDA-approved and has been shown to be generally well tolerated, we did not record adverse events or medication side effects. Additionally, other than ED visits, follow up with substance use services was not recorded. There was generally poor follow-up in our hospital’s addiction medicine clinic of approximately 5% (personal correspondence). Similarly, this study does not elucidate whether the changes in ED visit rates were due to naltrexone’s effect on alcohol consumption or other factors. We did exclude subjects who were admitted to the hospital during the 30 days after the index visit; however, there could have been admissions to hospitals outside our healthcare system.

We did not use a formal diagnosis of AUD when patients were offered naltrexone. Our chart flag was purposefully broad (at least one *ICD-10* code for an alcohol-related disorder in five years). We likely included many subjects who did not meet AUD criteria; perhaps this is why our total population showed no difference with naltrexone.

We chose four or more visits in the prior 90 days to define subjects with high ED use. While there is no accepted standard of how many visits constitute a patient with high use, typical definitions use the number of four or more visits annually.^30^,^31^ We specifically chose the 90-day time frame to account for the 30-day study period immediately after that 90 days so as not to include patients whose frequent visits may have occurred earlier. We wanted to include patients who were *currently* using the ED frequently before the index naltrexone visit. Therefore, our study population may differ from other studies with subjects with high ED use.

These limitations aside, there is promise in this study to suggest that naltrexone may be associated with reduced ED recidivism in certain patients, particularly those who make frequent ED visits. The implications could have a significant impact in reductions in the burden of alcohol use disorder on healthcare expenses and ED use.

## CONCLUSION

Naltrexone administered in the ED was associated with reduced ED recidivism in subjects with high ED use, but it had no associated change on the entire study population.

## Figures and Tables

**Table 1:** Demographics of index visits by patients who were not frequent users of the emergency department versus those who did present frequently.

	All visits (N = 331)	Visits by subjects who did not have high ED use^1^ (n = 275)	Visits by subjects who did have high ED use^2^ (n = 56)	*P* value
	n (%)^3^ or median (IQR)	n (%)^3^ or median (IQR)	n (%)^3^ or median (IQR)	
Age	43 (34–55)	41 (33–54)	51 (42–57)	< .001
Sex				.33
Female	101 (30.5%)	87 (31.6%)	14 (25%)	
Male	230 (69.5%)	188 (68.4%)	42(75%)	
Race				.59
White	204 (61.8%)	166 (60.4%)	38 (67.9%)	
Black	51 (15.5%)	44 (16%)	7 (12.5%)	
Other	75 (22.7%)	64 (23.3%)	11 (19.6%)	
Ethnicity				.86
Hispanic or Latino	79 (24.1%)	65 (23.6%)	14 (25%)	
Not Hispanic or Latino	249 (75.9%)	207 (75.3%)	42 (75%)	

1 More than 4 visits in the 90 days preceding the index naltrexone visit;

2 Fewer than 4 visits in the 90 days preceding the index naltrexone visit;

3 Percentage of each demographic category.

*ED*, emergency department; *IQR*, interquartile range.

**Table 2:** Number of visits in the 30 days prior and the 30 days following the naltrexone index visit among all visits, comparing visits by patients who did not present frequently to the emergency department to those who were characterized as high users.

	30 days prior median visits	30 days following median visits	30 days prior mean visits	30 days following mean visits	*P* value

	median (IQR)	median (IQR)	mean (SD)	mean (SD)	
All Visits (N = 331)	0 (0–1)	0 (0–1)	1.04 (2.4)	0.99 (2.36)	.53
Visits by subjects who did not have high utilization of the ED^1^ (n = 275, 83.1%)	0 (0–0)	0 (0–0)	0.25 (0.53)	0.43 (1.34)	.02
Visits by subjects who had high utilization^2^ (n = 56, 16.9%)	5 (3,8)	3 (2,6)	4.91 (3.84)	3.73 (3.88)	.02

1 <4 visits in the 90 days preceding the naltrexone index visit;

2 ≥ 4 visits in the 90 days preceding the naltrexone index visit.

*ED*, emergency department; *IQR*, interquartile range; *SD*, standard deviation.

**Table 3:** Impact of naltrexone index visit on subsequent 30-day emergency department (ED) visits between subjects with high ED use and less frequent ED use (n = 331).

	Less frequent ED use (n = 275)^1^	High ED use (n = 56)^2^	*P* value

	n (%)	n (%)	
Change in 30-day ED visits			<.001
Increase	50 (18.2%)	13 (23.2%)	
No Change	191 (69.5%)	7 (12.5%)	
Decrease	34 (12.4%)	36 (64.3%)	

1 < 4 visits in the 90 days preceding the naltrexone index visit;

2 ≥ 4 visits in the 90 days preceding the naltrexone index visit.

*ED*, emergency department.
